# The Impact of Dysmetabolic Sarcopenia Among Insulin Sensitive Tissues: A Narrative Review

**DOI:** 10.3389/fendo.2021.716533

**Published:** 2021-11-10

**Authors:** Angelo Armandi, Chiara Rosso, Gian Paolo Caviglia, Davide Giuseppe Ribaldone, Elisabetta Bugianesi

**Affiliations:** Department of Medical Sciences, Division of Gastroenterology and Hepatology, A.O. Città della Salute e della Scienza di Torino, University of Turin, Turin, Italy

**Keywords:** sarcopenia, insulin resistance, obesity, NAFLD, leptin, microbiota, irisin, myostatin

## Abstract

Sarcopenia is a common muscular affection among elderly individuals. More recently, it has been recognized as the skeletal muscle (SM) expression of the metabolic syndrome. The prevalence of sarcopenia is increasing along with visceral obesity, to which it is tightly associated. Nonetheless, it is a still underreported entity by clinicians, despite the worsening in disease burden and reduced patient quality of life. Recognition of sarcopenia is clinically challenging, and variability in study populations and diagnostic methods across the clinical studies makes it hard to reach a strong evidence. Impaired insulin activity in SM is responsible for the altered molecular pathways and clinical manifestations of sarcopenia, which is morphologically expressed by myosteatosis. Lipotoxicity, oxidative stress and adipose tissue-derived inflammation lead to both alterations in glucose disposal and protein synthesis in SM, with raising insulin resistance (IR) and SM atrophy. In particular, hyperleptinemia and leptin resistance interfere directly with SM activity, but also with the release of Growth Hormone from the hypohysis, leading to a lack in its anabolic effect on SM. Moreover, sarcopenia is independently associated to liver fibrosis in Non-Alcoholic Fatty Liver Disease (NAFLD), which in turn worsens SM functionality through the secretion of proinflammatory heptokines. The cross-talk between the liver and SM in the IR setting is of crucial relevance, given the high prevalence of NAFLD and the reciprocal impact of insulin-sensitive tissues on the overall disease burden. Along with the efforts of non-invasive diagnostic approaches, irisin and myostatin are two myokines currently evaluated as potential biomarkers for diagnosis and prognostication. Decreased irisin levels seem to be potentially associated to sarcopenia, whereas increased myostatin has shown to negatively impact on sarcopenia in pre-clinical studies. Gene variants in irisin have been explored with regard to the impact on the liver disease phenotype, with conflicting results. The gut-muscle axis has gain relevance with the evidence that insulin resistance-derived gut dysbiosis is responsible for increased endotoxemia and reduction in short-chain free fatty acids, directly affecting and predisposing to sarcopenia. Based on the current evidence, more efforts are needed to increase awareness and improve the management of sarcopenic patients.

## Introduction

During the last years, sarcopenia has been progressively recognized as the muscular expression of the metabolic syndrome (Mets), with relevant implications in both the pathophysiological field and in clinical setting. The compresence of diverse metabolic-related affections worsens the global disease burden, with a reciprocal impact on tissue metabolisms and clinical outcomes. Nonetheless, sarcopenia is still underestimated and underreported in patients with MetS. Therefore, the aim of the present review is to elucidate the molecular pathways involved in the crosstalk between insulin-sensitive tissues with respect to the onset and progression of sarcopenia, and how they translate into current clinical studies and future perceptive for clinical management. In addition, the major controversial points in the field will be discussed, pointing out the unmet needs that would require further investigation (**Table 1**).

**Table 1:** Unmet needs in the setting of sarcopenia that require further investigations.

Definition	Sarcopenia among individuals with metabolic syndrome represents a separate entity from that occurring in ageing population. Is s concomitantly found with obesity (“sarcopenic obesity”) and/or other features of metabolic syndrome. Obesity by Body Mass Index may be under-classified in patients with sarcopenia. A proper definition of sarcopenia and the understanding of all concomitant diseases and afflictions (e.g. depression) needs to be carefully implemented.
Diagnosis	Comparative studies involving the different diagnostic tools (hand-grip strength, gait speed) and techniques (Computed Tomography, Magnetic Resonance, Body impedance analysis, Dual-energy X-ray absorptiometry, ultrasound) are needed, in order to assess their accuracy in different populations (age, ethnicity) and to improve non-invasive, radiation-free approaches.
Biomarkers	Sarcopenia is a major determinant for the metabolic status in individuals with metabolic syndrome and insulin resistance. Many involved tissues (adipose tissue, liver, hypothalamus) actively secrete cytokines that might be feasible as biomarkers, as emerged by pre-clinical studies and few human studies (leptin, irisin, myostatin, adiponectin, IGF-1). Their plausibility is affected by the different source of secretion and the pleiotropic effect of the molecules. More studies are needed to assess the accuracy of these cytokines as serum markers in specific populations (e.g. individuals with Non-Alcoholic Fatty Liver Disease, diabetic patients) for the detection of sarcopenia and the prediction of a more severe course.
Therapy	The management of sarcopenia resides on physical activity. The concomitant presence of other conditions (e.g. older age, obesity) and potential lack of long-term compliance requires other approaches. The evaluation of multiple cross-talks between insulin sensitive tissues and different disease pathways might bring to light optimal target for individualized therapy.

## Linking Sarcopenia to Insulin Resistance

Insulin exerts major metabolic and anabolic effects upon the skeletal muscle (SM). Accounting for about 40-50% of the total lean mass, SM is responsible for the 80% of post-prandial glucose disposal, playing a crucial role in maintaining the whole body energy homeostasis. In addition, insulin promotes protein synthesis and limits protein catabolism, contributing to the trophism and physiology of myocytes. Impaired insulin action in SM in the setting of insulin resistance (IR), affects both glucose metabolism and the maintenance of a proper muscle mass. Conversely, a reduced SM functionality worsens IR and contributes to the metabolic abnormalities of the MetS. The impoverishment of muscle mass, or “sarcopenia”, refers to an unintentional weight loss, weakness and slowing in daily activities, mostly observed among elderly individuals (1). This process seems to start around the third decade of life and becomes increasingly relevant with ageing. In fact, sarcopenia is a major determinant of frailty in the elderly population, significantly contributing to all cause morbidity and mortality (2–4).

In recent years, the concept of a defective muscle mass has gained greater attention for its putative role in the cross talk of insulin sensitive tissues in subjects with MetS (5). The increasing prevalence of obesity worldwide has led to a concomitant increase in a phenotype currently defined as “sarcopenic obesity” (SO), predicted to affect up to 100-200 million subjects in the next 30 years (6), with a synergic amplification of disease burden (7). A recent meta-analysis reported a 24% increase in the risk of all-cause mortality among SO individuals, regardless of geographical distribution (8). In particular, male gender is addressed as a predictor for sarcopenia (9). Despite the combination of sarcopenia and weight gain is most commonly observed among the elderly (10), the additional harmful impact of SO in younger individuals is leading to an increase in overall mortality in the age range 50-70 (11, 12).

The obesogenic environment is promoted by sedentary lifestyle and improper calorie intake, either quantitative or qualitative. Notably, the hypernutrition observed among obese individuals is essentially a form of malnutrition that can directly affect the muscle mass because of the reduced intake of protein-based nutrients, in favor of refined carbohydrates, high glycemic index foods, and saturated fats. A prompt detection of sarcopenic individuals will acquire progressive relevance in the comprehensive management of the metabolic syndrome.

In addition, the definition of obesity itself, as defined by Body Mass Index (BMI), has some limitations when considering mobility impairments. A large study conducted on 852 adult individuals (852 had reported at least one physical disability and 4724 without impairments) showed that patients with functional mobility impairments did not fall into the obesity definition according to BMI (≥ 30 kg/m^2^), but were obese according to waist circumference and/or percentage of body fat detected by Dual-energy X-ray absorptiometry (DEXA). The impoverishment of muscle mass is likely to impact on BMI, which in this population might be misleading (13).

### Skeletal Muscle Metabolism: Insulin-Activated Pathways and Energy Sensors

In SM, insulin binds its tyrosine-kinase receptor to exert different actions, with respect to glucose metabolism and protein synthesis. Auto-phosphorylation of the receptor leads to recruitment of insulin receptor substrate (IRS)-1, which guides downstream pathways.

Activation of phosphatidylinositol 3-kinase (PI3K) promotes phosphorylation of protein kinase B (PKB)/AKT and allows glucose internalization by translocation of glucose transporter (GLUT)-4 from vesicles to plasma membrane. Glycogen synthesis is stimulated by phosphorylation of glycogen synthase kinase 3 (GSK3). All these actions aim at glucose disposal and storage.

In addition, PKB/AKT activates the mammalian target of rapamycin (mTOR), 4E-binding protein 1 (4E-PB1) and ribosomal S6 kinase 1 (S6K1), involved in the protein synthesis, important for muscle mass anabolic metabolism and trophism.

Another key signaling pathway is represented by the AMP-activated kinase (AMPK), that promotes glucose and FFA uptake/metabolism and modulates long-term responses in mitochondria, by interacting with peroxisome proliferator receptor gamma activator 1α (PGC-1α) (14). In the presence of intracellular energy deficiency, AMPK inhibits protein synthesis by suppressing mTOR signaling (15).

Sensitivity to insulin varies across different types of muscle fibers. Muscle oxidative metabolism is prevalent in Type I fibers, richer in mitochondria, whereas glycolytic pathways mostly occur in type II fibers. Enhanced oxidative capacity in type I fibers is linked to a better responsiveness to insulin and its anabolic effect. In individuals with MetS, type I fibers are less abundant according to the severity of IR, concurring to the development of sarcopenia (16).

### Sarcopenia: Assessment and Clinical Implications

An appropriate evaluation of sarcopenia is of crucial relevance in clinical studies in order to minimize heterogeneity and to address a proper intervention.

Easy first-line assessments such as handgrip strength (17), or short endurance performances like gait speed or the chair test, may raise suspicion of SM impairment but are not considered fully reliable due to lack of accuracy and standardizations (18). Nonetheless, they are recommended as quick and safe tests to guide clinicians towards more accurate examinations.

Imaging parameters obtained by computed tomography (TC) or magnetic resonance imaging (MRI) are currently considered the gold standard, despite several limitations due to costs, availability and radiation exposure (19).

DEXA is the most reliable method for evaluation of SM. According to the European Working Group on Sarcopenia in Older People (EWGSOP), identification of sarcopenia requires the DEXA assessment of the appendicular SM mass (ASM) to calculate the skeletal mass index (SMI) by the formula: ASM/height^2^. A value below two standard deviations from reference defines sarcopenia (5). However, this score is appropriate for lean people, but underestimates the entity of sarcopenia among SO individuals. Hence, the modified index ASM/weight has been proposed to better quantify sarcopenia across metabolic disturbances (20). Alternatively, the ASM/BMI index has been evaluated in Korean populations, where it would be more tightly associated to IR and visceral obesity (21). Body impedance analysis (BIA) has been proposed as a valuable surrogate for the favorable cost-effective profile and avoidance of radiations, despite lack of validation in severely obese individuals and potential interference of hydration status (5).

In addition, SM ultrasound might be a useful first-line tool for sarcopenia assessment, in particular among special populations, as bedside examination. In recent times, ultrasound of the quadriceps has shown acceptable reliability in detection muscle quantity and explore muscle quality, even when edema or fluid retention are present (22).

In clinical setting, a comprehensive evaluation of patients with sarcopenia would require an evaluation of quality of life. Motility impairment is a source of both physical and physiological affliction. In fact, depressive symptoms have been associated to sarcopenia, in particular among elderly individuals (23). This might be linked to the cognitive environment in ageing populations, but can also depend on the systemic proinflammatory status promoted by MetS that represents a pathophysiology milieu for depression as well. In fact, depression is also highly prevalent among individuals with hepatic steatosis, which is the liver hallmark of MetS (24). Patients reported outcomes represent a crucial step in the clinical evaluation and depressive symptoms should not be overlooked in this population.

## Pathophysiology of Dysmetabolic Sarcopenia

### Myosteatosis Causes Impairment in Muscle Function

Excessive intramyocellular lipid infiltration, known as “myosteatosis”, play a crucial role in the impairment of muscle function in the setting of systemic IR. In IR states, the spillover of free fatty acids (FFA) from a dysfunctional and inflamed adipose tissue into ectopic sites, together with the persistent hyperinsulinemia and hyperglycemia, lead to organ-specific lipotoxicy and glucotoxicity. In the pancreas, this favors the onset of type 2 diabetes mellitus (T2DM). In the liver, this can lead to NAFLD, ranging from simple steatosis (Non-Alcoholic Fatty Liver, NAFL), to a progressive inflammatory disease (Non-Alcoholic Steatohepatitis, NASH), with deposition of fibrotic tissue that leads to cirrhosis and end-stage disease (25). In SM, a worsening in glucose uptake and in FFA oxidation may further impair glucose homeostasis and protein synthesis, leading to sarcopenia (26).

One seminal study, conducted on old mice fed with high fat diet for 10 weeks, connected the reduced ability to store lipids inside the adipose tissue to an increased lipid deposition in SM and a decreased intramuscular protein synthesis (27). Fatty acid intermediates, like ceramides or diacylglycerol, are involved in the impairment of insulin signaling (28), as they interfere on the nucleus-mitochondrial crosstalk (29). Both mice and humans undergoing lipid infusion show intramuscular increase in diacylglycerol and activation of two isoforms of protein kinase C (PKC) (δ and θ), which negatively regulate insulin receptor activity (30). The excessive lipid deposition leads to mitochondrial activity overload, reduction of β-oxidation of FFA and increase in both long and short-chain acylcarnitine species; in turn this increases oxidative stress and impairs PKB/AKT phosphorylation (31), reducing glucose utilization and glycogen synthesis (32). Additionally, saturated fat-derived ceramide accumulation directly impairs muscular protein synthesis, enhancing phosphorylation of eukaryotic initiating factor 2 α (eIF2α) (27), and protein translation by suppressing factor 4E-BP1 phosphorylation (33).

Lipid infiltration also directly acts by progenitors of adipocytes in myotubes, which exert a paracrine effect on SM function, impairing insulin-mediated glycogen synthesis and glucose uptake as shown by a reduced PKB/AKT phosphorylation (34, 35). These evidences highlight the putative role of lipotoxicity in driving time-dependent muscle atrophy (36); on the other hand, the latter contributes to worsening of peripheral IR.

### Impact of Visceral Adipose Tissue and Leptin Resistance

**Figure 1:**
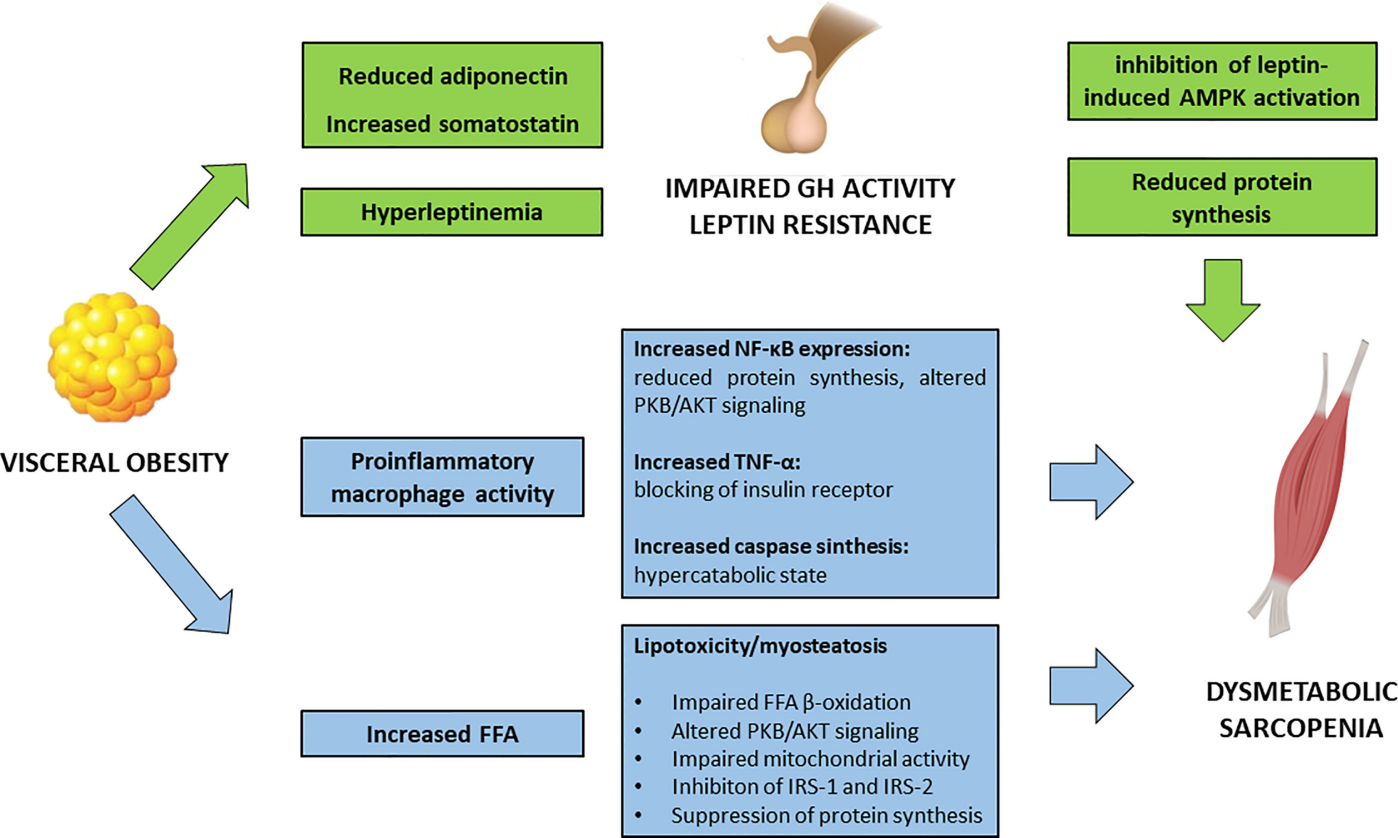
Impact of visceral obesity on sarcopenia. FFA, free fatty acids; GH, growth hormone; IRS, insulin receptor substrates; NF-κB, Nuclear Factor – κB; PKB/AKT, Protein kinase B/AKT; TNF-α, Tumor Necrosis Factor – α.

Visceral obesity is a pathological condition where the adipose tissue represents an actively secreting organ, contributing to a pro-inflammatory condition (**Figure 1**). In fact, rather than a simple excessive fat accumulation, the expansion of the adipose tissue, in terms of both hypertrophy and hyperplasia, is accompanied by an increase in inflammatory cell types. Activated macrophages in the adipose tissue release several pro-inflammatory cytokines enhancing local and systemic inflammation.

*In vitro* studies have shown how macrophages, in an obesogenic mimicking environment, are able to infiltrate the SM and to interfere with muscle function, by decreasing Nuclear Factor (NF)-κβ inhibiting protein [Inhibiting κβ-α (Iκβ-α)] and phosphorylated PKB/AKT (37). In particular, NF-κβ has a pleiotropic effect in the muscle, and its activation is associated with a decrease in protein synthesis and an increase in protein degradation, leading to reduction in muscle strength and atrophy (38). Moreover, murine models have shown that an obesogenic diet leads to a significant increase in ubiquitin-proteasome pathways and caspase synthesis in SM, related to an accelerated catabolic activity (39).

Accordingly, human observational studies showed that obesity represents a significant risk factor for the development of sarcopenia (40). Leptin, secreted by adipose tissue, acts as a pro-inflammatory hormone; serum levels of leptin are higher in subjects with SO, rather than in those with either sarcopenia or visceral obesity alone (41). Hyperleptinemia is also the result of leptin resistance and defective signaling at the hypothalamic neurons (42, 43).

In healthy individuals, leptin stimulates AMPK in SM. In obese subjects, this pathway is suppressed, and this is thought to be partly attributed to the increased hypothalamic expression of the obesity-related suppressors of cytokine signaling 3 (SOCS3). In rodents, SOSC3 inhibits leptin activation of AMPK, contributing to the impaired fatty acid metabolism in SM (44).

### Impact of Growth Hormone and Adipokines

The detrimental impact of visceral adipose tissue in muscle function partly resides on its interference on the hypothalamus-hypophysis axis of Growth Hormone (GH)/Insulin-like Growth Factor-1(IGF-1) and Growth Hormone Releasing Hormone (GHRH). GH exerts a trophic effect on SM, enhancing protein synthesis and β-oxidation of FFA. Obesity is associated with a reduced GH activity, which seems to be a functional deficiency and potentially reversible with weight loss (45). In particular, obesity-specific endocrine alterations significantly contribute to the impairment in GH activity: decreased adiponectin levels, an adipokine that exerts anti-inflammatory and anti-fibrotic effect, and increased somatostatin, are the main drivers of GH deficient action. In addition, obesity-related hyperinsulinemia and elevated FFA are key modifiers of GH release. Defective GH signaling in the visceral adipose tissue contributes to impaired activity of the hormone-sensitive lipase, that furtherly promotes fat accumulation (46).

Leptin can exert a negative regulation on GH secretion in obese humans *via* suppression of hypothalamus signaling (47).

Adipocyte fatty acid-binding proteins (FABP) is another adipokine that may play a role in SO. FABP4 is expressed mainly in adipocytes and macrophages, and has been used as marker of adipose tissue differentiation. FABP4 binds FFA with high affinity and is involved in the regulation of intracellular FFA trafficking among various cellular compartments. Furthermore, FABP4 shuttles several molecules into the nucleus enhancing gene transcription. Knockout mice for FAPB4 are protected from the onset of insulin resistance and obesity (48), and studies on humans have highlighted the connection between increased levels of FABP4 and T2DM (49). In SM, FABP4 is higher in endurance-trained individuals compared to moderately active subjects, and favors the trafficking of FFA towards the mitochondria (50). However, in cross-sectional studies conducted on SO individuals, increased levels of FABP-4 were independently associated to DEXA-proven sarcopenia, making this adipokine a potential marker of muscle deficiency among obese individuals (51).

The pro-inflammatory adipokine resistin can interfere with insulin signaling by activation of SOCS3 (52). In mouse models, an association between increased levels of resistin and sarcopenia has been found (53). Another adipokine, the transporting retinol-binding protein (RBP)-4, is increased in the setting of adipose tissue insulin resistance, where it enhances pro-inflammatory pathways upon the macrophages and impairs adipocyte function *via* paracrine signaling. In murine models, increased RBP-4 seems to cause a direct interference of insulin action inside the SM, potentially contributing to sarcopenia (54).

In addition, SO is associated with higher levels of interleukin (IL)-6 and C-reactive protein (CRP), further highlighting the link between the adipose tissue-derived systemic inflammation and impaired SM functionality (55, 56). Moreover, high levels of macrophage-derived Tumor Necrosis Factor (TNF)-α directly contributes to IR, by blocking insulin receptor upon the SM (57).

Future evaluation of the cytokines pattern in the setting of metabolic dysfunction would provide insightful evidence on sarcopenia and help on risk stratification.

### Sarcopenia in the Cross-Talk Between Adipokines and Hepatokines

**Figure 2:**
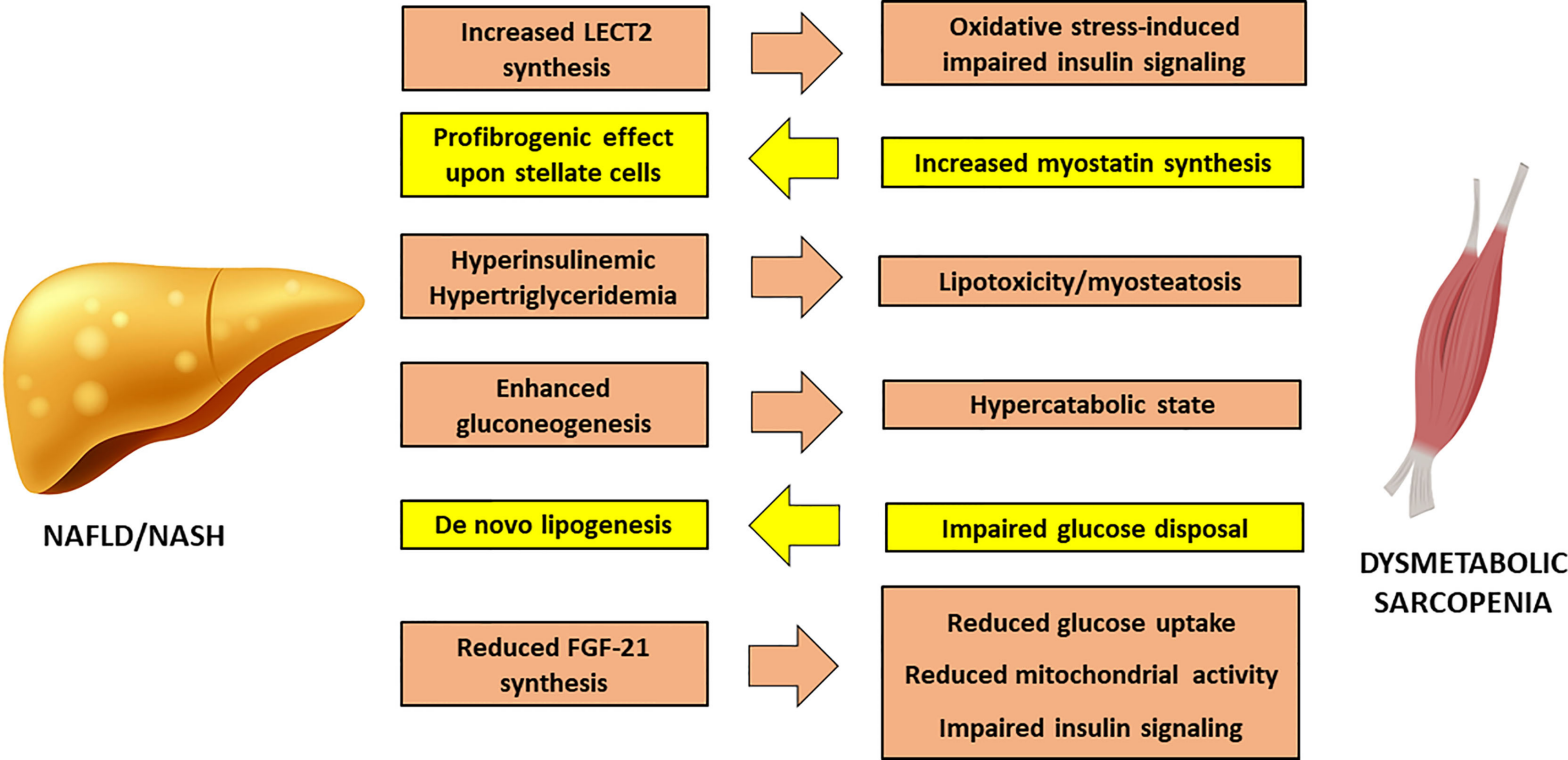
Cross-talk between Non-Alcoholic Fatty Liver Disease and sarcopenia. FGF-21, Fibroblast Growth Factor – 21; LECT2, Leucocyte cell-derived chemotaxin 2; NAFLD, Non-Alcoholic Fatty Liver Disease; NASH, Nonalcoholic Steatohepatitis.

Non-Alcoholic Fatty Liver Disease (NAFLD) may develop in IR-states as a consequence of increased FFA delivery to the liver from a dysfunctional adipose tissue and increased *de novo* lipogenesis sustained by hyperinsulinaemia. A fatty liver overproduces very-low density lipoproteins (VLDL), thus further contributing to myosteatosis and sarcopenia (**Figure 2**).

In turn, SM IR can worsen liver steatosis through direct glucotoxicity, as shown by studies conducted on knockout mice for SM-restricted GLUT4 (58), where undisposed glucose is addressed to hepatic *de novo* lipogenesis, increasing intracellular lipid load. In addition, enhanced gluconeogenesis in the liver causes a persistent catabolic state of SM, in order to supply the liver with the protein-derived aminoacids as substrate for glucose synthesis, thus exacerbating sarcopenia.

The chronic inflammation of the adipose tissue has a direct impact on liver injury, promoting the activation of Kupffer cells (59). Reduction in adiponectin worsens hepatic insulin sensitivity and favors fat accumulation in liver parenchyma (60). The concomitant hyperleptinemia exerts a direct effect on hepatic stellate cells, that promote fibrogenesis, and enhances the synthesis of transforming growth factor (TGF)-β through a direct effect upon Kupffer cells (61). Moreover, obesity is associated to a reduction in hepatic synthesis of Fibroblast Growth Factor (FGF)-21, that stimulates glucose uptake, mitochondrial activity and thermogenesis. In liver and SM, FGF-21 improves diacylglycerol levels and inhibits PKC translocation (62), providing a mechanistic link between its reduced activity and the onset of IR.

Proinflammatory hepatokines can exert a direct action on the SM. Leucocyte cell-derived chemotaxin 2 (LECT2) is a cytokine that positively correlates to SM IR and obesity. LECT2 induces Jun N-terminal kinase (JNK) phosphorylation in myocytes, leading to impairment of insulin sensitivity in mice (63). Observational studies conducted on humans have shown that significantly higher levels of LECT2 are found in patients with NAFLD and MetS (64).

Fetuin A is another hepatokine that affects insulin sensitivity by inhibition of insulin receptor autophosphorylation (65); in addition, it has shown a good association with NAFLD in a recent systematic review and meta-analysis of 1755 patients and 2010 healthy controls (66). Fetuin A seems to impact on body composition among older individuals, favoring accumulation of visceral adipose tissue (67), and a positive association with sarcopenia has also been reported (68). Finally, Selenoprotein P, an hepatokine that has been associated to a deranged metabolic profile and to the worsening of liver disease in NAFLD patients (69), inhibits AMPK inside the liver, favoring IR. Interestingly, one recent study has proposed a new model for prediction of sarcopenia in mice based on four molecules, including Seleoprotein P. This model resulted significantly associated to a high risk of developing sarcopenia, linking liver pathophysiology to defective muscle regeneration (70).

### Sarcopenia and NAFLD: Two Entangled Entities

NAFLD affects one quarter of individuals worldwide, representing a significant burden on health systems. In this context, SO is clinically relevant for patient’s management and prognostication. One Italian study conducted by Petta et al. on 225 patients with biopsy-proven NAFLD showed that prevalence of sarcopenia, identified by BIA-derived SMI, increased linearly with the severity of liver fibrosis and resulted independently associated with severe fibrosis (OR 2.36, p = 0.01) and steatosis (OR 2.02, p = 0.03) (71). In one Asian study, sarcopenia, identified by CT-derived categorical indices of both low SM mass and evidence of myosteatosis, resulted an independent prediction of significant liver fibrosis (OR 2.17, p < 0.05) (72). In another study in Korean population, the lowest quartile of DEXA-derived SMI was independently associated with the risk of NAFLD, albeit with less strong significance (OR 5.16, p = 0.041) (73).

Shared metabolic abnormalities can partially explain the link between NAFLD and SM. However, the independent association between the liver and the SM highlights the reciprocal interference on the overall disease burden. One cross-sectional population-based study from Korea showed that DEXA-based evidence of sarcopenia was associated to an increased risk of NAFLD regardless of obesity (OR 3.02, p < 0.001) or MetS (OR 4.00, p < 0.001), which are the strongest drivers of dysmetabolic diseases both in SM and in liver (74). Subsequent studies conducted on biopsy-proven NAFLD patients confirmed the association of sarcopenia with significant liver fibrosis (OR 2.05, p < 0.05) (75). Accordingly, one 7-year longitudinal study showed that the increase in BIA-based SM mass was associated with a reduced incidence of NAFLD (HR 0.44, p < 0.001) and with resolution of baseline NAFLD (HR 4.17, p < 0.001) (76).

Genetic predisposition could play a role both in NAFLD and sarcopenia. Variants in gene encoding for patatin-like phospholipase domain-containing 3 (*PNPLA3*) have been associated with an increased incidence of fat accumulation, liver inflammation and fibrosis, and hepatocellular carcinoma (HCC). One study explored the potential risk of sarcopenia among carriers of *PNPLA3* variants; DEXA-derived ASM independently decreased in NAFLD patients carrying the wild type gene, but no association was found among *PNPLA3* subjects who carried the risk allele (77).

Additional genetic, investigations have been carried out on polymorphisms in fibronectin type II domain-containing protein 5 (*FNDC5*), a myocyte membrane protein that is cleaved and released in the bloodstream as irisin. Irisin is an exercise-induced myokine, involved in the thermogenesis and browning of adipose tissue by stimulation of uncoupling protein (UCP)-1, able to reduce fat accumulation (78). Irisin can also improve IR by enhancing GLUT4 translocation and β-oxidation of FFA *via* energy sensor AMPK (79). Irisin serum levels are associated with SM mass, increasing along with exercise training (80). Accordingly, a cross-sectional study has shown a mild, but significant association between low levels of irisin and sarcopenia (OR 0.2, p < 0.01); interestingly, a cutoff of < 1 µg/ml predicted sarcopenia with an acceptable area under the receiver operating characteristic curve (AUROC) of 0.87 (81). Nonetheless, other evidences from literature failed to attribute a significant role to irisin in discriminating between sarcopenic and non-sarcopenic individuals (82) and a clear role of irisin in sarcopenia is still under debate.

One study described that rs3480 polymprohism in *FNDC5* gene was associated with severe steatosis in NAFLD population, through microRNA epigenetic control of irisin stability, and conversely increased levels of irisin among wild type carriers were associated to reduced steatosis and a better metabolic profile (83). Another study reported a protective effect of the gene variant on advanced liver fibrosis (84), but the prevalence of sarcopenia and liver histologic features is not different across the different genotypes of *FNDC5* polymorphism (85).

These differences may be partly explained by the heterogeneity of the study populations and the different methods used for sarcopenia assessment. However, the multifactorial genesis of sarcopenia can be also responsible for the protean phenotype (86).

The pathophysiology of NAFLD is still under investigation around the preeminent role of insulin resistance, and this aspect is translated into the current management of this liver disease, which lacks pharmacological therapies and resides on lifestyle modifications (87). Physical exercise is considered part of the cornerstone for NAFLD improvement, and this aspect would give major benefits on SM metabolism accordingly.

### Myostatin and Myokines Between Sarcopenia and Insulin Resistance

Myostatin, a myokine belonging to TGF-β superfamily, regulates SM metabolism both in an autocrine and paracrine way, and exerts endocrine activity upon peripheral tissues. Myostatin is stimulated by physical inactivity and favors fat deposition by decreasing adiponectin levels, with subsequent reduction in fat oxidation and increase in liver steatosis (88). Animal studies conducted on myostatin-deficient mice have shown that the impairment of insulin signaling is exerted through suppression of AMPK and PKB/AKT pathways (89). Moreover, myostatin is a negative key regulator of lipolysis and thermogenesis (90). Consistently, old mice treated with anti-myostatin antibodies displayed an increase in SM mass and strength, and a better glucose uptake measured by hyperinsulinemic-euglicemic clamp (91). Human studies have shown an enhanced transcription of myostatin in sarcopenic individuals, interfering with the anabolic GH/IGF-1 pathway (92). It can be speculated that leptin-derived impairment of GH activity may lead to a reduced suppressive action of GH on myostatin, favoring its increased activity.

Interestingly, myostatin receptor (activing-receptor-2B) is upregulated in mouse fibrotic livers, and detected on human hepatic stellate cells. Myostatin modulates JNK pathway, enhancing cell migration and expression of procollagen type 1 and TGF-β1, thus favoring a profibrogenic phenotype (93). This finding may represent a further evidence of interplay between different tissue alterations and physiopathological pathways.

These evidences also highlight a potential role of myostatin inhibition as therapeutic target to treat sarcopenia. Moreover, in the last years multiple pre-clinical studies have reported a large number of myokines involved in onset of sarcopenia, elucidating an increasing complexity in the cross talk between insulin-sensitive tissues. Myokines, and more generally cytokines, are expressed by diverse tissues, with different roles and biological plausibility. Leukemia inhibitory factor (LIF), for instance, is an exercise-induced myokine, involved in the SM biogenesis, that acts in a autocrine way and is mostly undetectable in serum. Nonetheless, LIF is also majorly synthetized by cancer cells to induce cachexia and potentially a target to treat SM atrophy in this population (94). Angiopoietin-like 4 (ANGPTL4) is another example of the pleiotropy of these molecules: it is synthesized by adipose tissue and SM in response to fasting or hypoxia, aiming at maintaining body weight and inhibiting lipoprotein lipase. Gene variants of ANGPLT4 causing a reduce cytokine function have shown to improve glucose tolerance, suggesting a potential role as therapeutic target in T2DM individuals (95). Therefore, more studies are needed to investigate therapeutic targets to bring the mechanistic pre-clinical evidence into human applications.

### The Gut-Muscle Axis: Implications of Aminoacids and Short-Chain Fatty Acids

**Figure 3:**
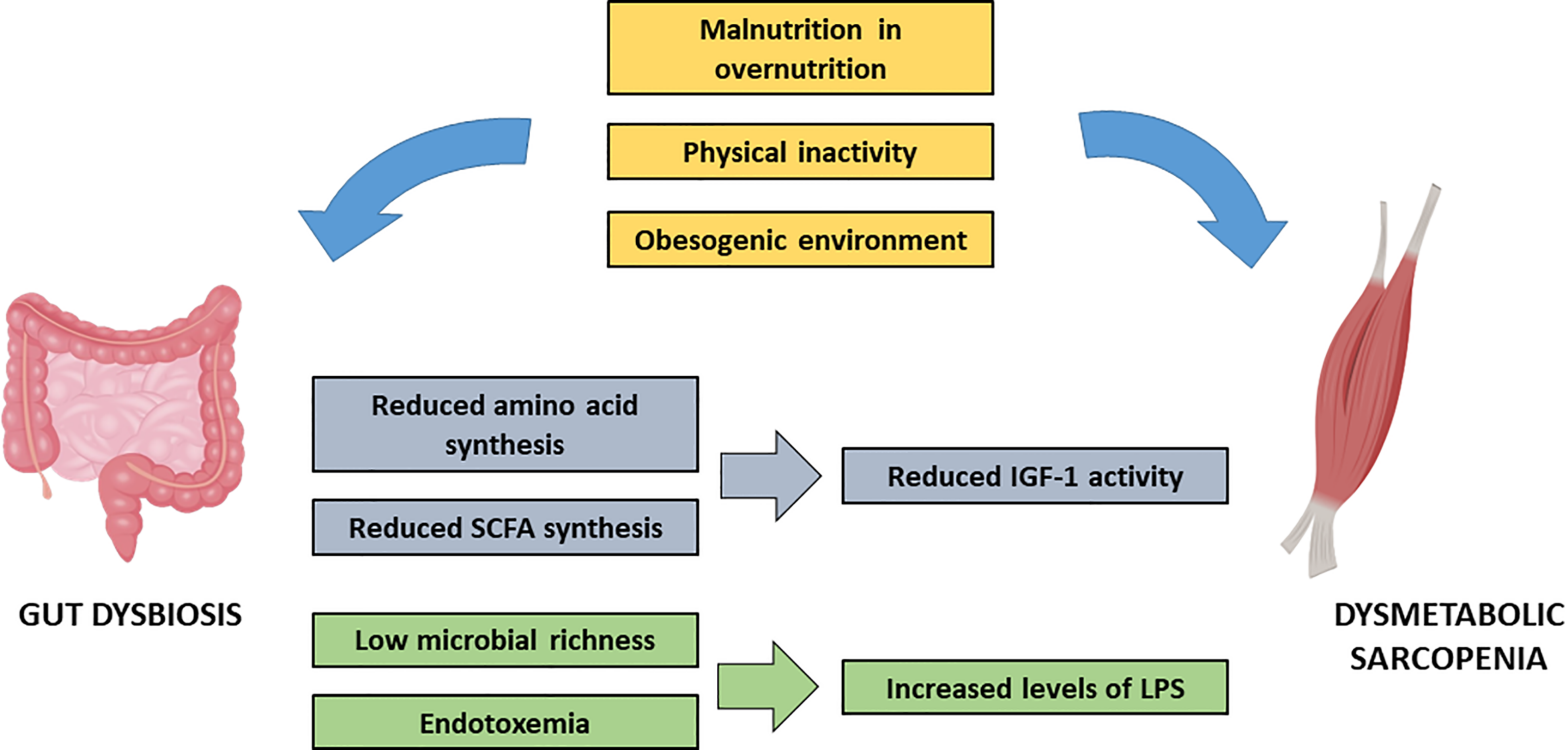
Impact of environmental factors and gut insulin-resistance derived gut dysbiosis on sarcopenia. IFG-1, Insulin-like Growth Factor-1; LPS, lipopolysaccharide; SCFA, short chain fatty acids.

Multiple environmental agents may be responsible for SM vulnerability. Malnutrition and physical inactivity are the most important factors that favor sarcopenia in the context of metabolic syndrome. More recently, the existence of a gut-muscle axis has been hypothesized, following the increasing evidence of a role of gut microbiota in the setting of both SM alterations and in IR (**Figure 3**). Gut microbiota metabolizes exogenous proteins and synthesizes essential aminoacids, like tryptophan, which is relevant for SM anabolism (96). Mice undergoing tryptophan supplementation show significant increase in IGF-1 and in the myostatin antagonist follistatin. A parallel improvement in protein synthesis *via* upregulation of mTOR pathway is observed (97).

Short-chain fatty acids (SCFA) are the end-products of gut microbiota anaerobic fermentation, produced mainly by Firmicutes species. SCFA provide multiple effects on energy metabolism (98), by binding of different receptors in peripheral tissues. In particular, G protein-coupled receptor 43 (GPR43) acts as a sensor of excessive dietary energy, regulating metabolic homeostasis (99). Knockout mice for GPR43 are obese under normal diet, whereas overexpression of the receptor leads to inhibition of fat accumulation. In a mouse model of T2DM, exercise training reversed the reduction in intestinal and plasma SCFA and improved SM IR by inducing muscle cell autophagy (100). Moreover, SCFA are key modulators of IGF-1 synthesis (101), which may provide a further link between SM metabolism and gut microbiota.

In particular, butyrate is a SCFA that is actively involved in SM metabolism. It limits protein degradation by inhibition of ubiquitin-proteasome catabolic pathway, enhances protein synthesis *via* mTOR pathway, and stimulates muscle stem cell differentiation *via* PI3K/AKT signaling (102), hence being one potential tool for detection and management of sarcopenic individuals.

Dietary intake and physical activity continuously shape gut microbiota, promoting different patterns of microbial species and modulating its heterogeneity. Obese people are characterized by low microbial richness, which favors inner imbalance among the species and the raise of pathogenic bacteria. Lipopolysaccharide (LPS), the main bacterial product of this disequilibrium, promotes a chronic systemic low-grade inflammatory activity (the so-called endotoxemia). By binding Toll-like receptor 4 (TLR4), LPS promotes IR by disrupting PI3K/AKT and NF-κβ pathways (103). One study conducted on old individuals showed an increased expression of TLR4 in SM, along with serum increase in LPS, associated to IR and to decreased quadriceps volume mass and muscle strength (104).

Therefore, SM functionality seems to be tightly dependent from lifestyle shaping of gut microbiota. Lifestyle interventions aiming at improving insulin sensitivity might result in a parallel improvement in SM through the gut-muscle axis.

## Conclusions

Despite the multifactorial origin of sarcopenia, ranging from ageing to chronic systemic inflammation, the onset of SM alterations in the setting of MetS requires unique considerations. Obesity as a result of unhealthy lifestyle, drives the systemic expressions of IR. In turn, metabolic imbalance in insulin sensitive tissues, mainly liver, SM and hypothalamus-hypophysis axis, contributes to the energetic homeostasis disruption and worsens systemic insulin sensitivity.

SM alterations that occur in IR states lead to a specific phenotype of sarcopenia, strongly linked to muscle IR. Reduction in glucose disposal and a reduced protein synthesis are the main consequences of sarcopenia, that furtherly impair IR and lead to a loss in muscle strength, frequently observed in obese individuals. Malnutrition associated with Western lifestyle, i.e. a reduction in proteins in favor of refined carbohydrates and saturated fats, impacts on SM health. Additionally, dysfunctional shaping in gut microbiota by unhealthy lifestyle actively contributes to SM impairment, while improving systemic IR and obesity through lifestyle interventions may be beneficial for SM as well.

Given the clinical implications, a comprehensive evaluation in patients with metabolic comorbidities should be advised, to allow a better risk stratification. Unfortunately, the high heterogeneity across study populations together with the different strategies used to diagnose sarcopenia, affects the quality of the results. DEXA is the most reliable and cost-effective tool to detect sarcopenia, and some efforts have been carried out to identify imaging-derived indexes which would be better applied across the different populations.

The need for non-invasive diagnosis of sarcopenia and for long-term implications have led to the evaluation of potential serum biomarkers, along with a diverse genetic susceptibility given by gene variants. Irisin and myostatin, for instance, have been studied with regard to diagnostic accuracy in discriminating sarcopenic patients, and to possible connections between sarcopenia and liver disease. NAFLD and sarcopenia share common pathophysiology pathways and have shown a strong association, regardless of other metabolic comorbidities. The impaired endocrine activity of both liver and SM has reciprocal implications and should not be overlooked in clinical setting.

Understanding the effective burden of insulin sensitive tissues in the complex picture of MetS has proved to be crucial in cross-sectional studies. However, longitudinal evaluations, with careful detection of study populations and designs, involving the potential role of non-invasive biomarkers, are still an unmet, yet urgent need.

## Author Contributions

AA: conceptualization and original draft preparation. EB: conceptualization and finalization of the manuscript. CR, GC, and DR: critical revision of the manuscript for intellectual content. All authors contributed to the article and approved the submitted version.

## Funding

Italian Ministry for Education, University and Research (Ministero dell’Istruzione, dell’Università e della Ricerca – MIUR) under the programme “Dipartimenti di Eccellenza 2018 – 2022” Project code D15D18000410001.

## Conflict of Interest

The authors declare that the research was conducted in the absence of any commercial or financial relationships that could be construed as a potential conflict of interest.

## Publisher’s Note

All claims expressed in this article are solely those of the authors and do not necessarily represent those of their affiliated organizations, or those of the publisher, the editors and the reviewers. Any product that may be evaluated in this article, or claim that may be made by its manufacturer, is not guaranteed or endorsed by the publisher.
